# Evaluation of the Adsorption Efficiency of Graphene Oxide Hydrogels in Wastewater Dye Removal: Application of Principal Component Analysis

**DOI:** 10.3390/gels8070447

**Published:** 2022-07-18

**Authors:** Omar Mouhtady, Emil Obeid, Mahmoud Abu-samha, Khaled Younes, Nimer Murshid

**Affiliations:** College of Engineering and Technology, American University of the Middle East, Kuwait; omar.mouhtady@aum.edu.kw (O.M.); emil.obeid@aum.edu.kw (E.O.); mahmoud.abusamha@aum.edu.kw (M.A.-s.)

**Keywords:** hydrogel, sustainability, wastewater treatment, principal component analysis, graphene oxide, adsorption, hydrogel composites, dye, machine learning, artificial intelligence

## Abstract

Industrial dye wastewater is one of the major water pollution problems. Adsorbent materials are promising strategies for the removal of water dye contaminants. Herein, we provide a statistical and artificial intelligence study to evaluate the adsorption efficiency of graphene oxide-based hydrogels in wastewater dye removal by applying Principal Component Analysis (PCA). This study aims to assess the adsorption quality of 35 different hydrogels. We adopted different approaches and showed the pros and cons of each one of them. PCA showed that alginate graphene oxide-based hydrogel (without polyvinyl alcohol) had better tolerance in a basic medium and provided higher adsorption capacity. Polyvinyl alcohol sulfonated graphene oxide-based hydrogels are suitable when higher adsorbent doses are required. In conclusion, PCA represents a robust way to delineate factors affecting hydrogel selection for pollutant removal from aqueous solutions.

## 1. Introduction

Dyes are used primarily in the production of consumer products, including paints, textiles, printing inks, paper, and plastics. Each year, the discharged dyes reach 60,000 tons worldwide. Dyes consist of synthetic organic material with biological toxicity such as carcinogenicity and teratogenicity and are mutagenic [1]. The main source of synthetic and organic dyes is the textile dyeing process. Azo dyes are the largest group of artificial dyes, corresponding to 65% of the total production of dyes in the world [2]. Synthetic dyes are refractory to temperature [3] and very stable due to their complex molecular structure and, therefore, do not biodegrade easily [4]. Consequently, dye-contaminated water discharged by industrial activities, including dye production, is one of the major water pollution problems posing a serious risk to drinking-water supplies [5].

Enormous efforts and various physical, chemical, and biological remediation approaches have been developed to treat the aquatic environment. However, physical methods, including adsorption, have shown promising and sustainable efficiency for treating dye-contaminated water [6]. Adsorbent materials are yet considered one of the most promising strategies to remove contaminants [7]. By definition, adsorption is a phenomenon of surface in which a solute (atom, ion, or molecule in a gas or liquid state) adheres to a solid sorbent. The advantages of adsorption processes are mainly their simple design, low cost, and their effectiveness towards a wide range of pollutants compared to other approaches (coagulation, filtration, precipitation, ion exchange, reverse osmosis, and oxidative processes) [8,9,10].

The tendency to favor better adsorption results is observed when the dye-contaminated waters, hydrophilic, and functional materials are taken into consideration. In recent years, studies have focused on using composite hydrogels for adsorption due to their promising properties compared to conventional hydrogels or other hydrophilic materials [6]. Hydrogels are three-dimensional networks of hydrophilic polymers that can absorb large amounts of water and swell while maintaining their structure due to the chemical or physical cross-linking of individual polymer chains [6]. These composites can be enriched with hydrophilic and functional groups, which enhance the adsorption of dyes and heavy metal ions from aqueous solutions.

Adsorbents and environmental applications of graphene-based composites have been reviewed for dye removal [8,11]. Activated carbon has been used intensively in dye manufacturing industries due to its sustainability and cost-effectiveness [12]. The new prospect of pollutant management is the combination of nanomaterials such as metal oxides, graphene, and carbon nanotubes. Graphene is massively used as a nano-adsorbent for environmental applications due to its high theoretical surface area (~2620 m^2^g^−1^) [13,14]. Graphene oxide (GO) is mainly produced from graphene by the Staudenmaier method [15]. GO has abundant oxygen-containing functional groups on its surface and can be processed into reduced graphene oxide (rGO) [16].

The surface functionality and electrostatic interactions of the adsorbate make GO a very promising material for environmental applications [17], such as the adsorption of charged species [18]. However, the efficiency of adsorption of GO depends on the charge on the dye [4].

To evaluate the adsorption efficiency of GO hydrogels in wastewater dye removal, Principal Component Analysis (PCA) with several parameters has been applied. In general, PCA is used to reduce the parameters of a dataset by producing linear combinations of the original parameters and, therefore, to identify the main parameters necessary to enhance and improve a given process [19].

Following the large number of parameters that affect the efficiency of GO for wastewater remediation, a PCA approach can be adopted to better seek intercorrelation in parameters related to adsorption efficiency. To the best of our knowledge, this work represents the first statistical and artificial intelligence study applied to evaluate the adsorption efficiency of GO hydrogels for dye removal.

## 2. Methodology

The aim of the study is to apply PCA based on the published study by Pereira et al. [6] (Table 1) to better understand the functional difference of multiple GO-based hydrogels depending on their adsorption properties. PCA is a method of revealing patterns among variables. These patterns were hidden from the bi-dimensional statistical approach. It presents an unsupervised machine-learning method since, once applied, no prior knowledge is assumed regarding the data or the investigated phenomena. The *j*th PC matrix (*Fi*) is expressed using a unit-weighting vector (*Uj*) and the original data matrix *M* with *m* × *n* dimensions. (*m*: number variables *n*: number of datasets) as follows [19,20,21,22]:

(1) $$Fi=U_{j}^{T}M=\overset{i=0}{\underset{}{\sum }}U_{ji}M_{i}$$

where *U* is the loading coefficient and *M* is the data vector of size *n*. The variance matrix *M*(*Var*(*M*)), which is obtained by projecting *M* to *U*, should be maximized, following:

(2) $$Var\left(M\right)=\frac{1}{n}\;\left(UM\right){\left(UM\right)}^{T}=\frac{1}{n}\;UMM^{T}U$$

(3) $$MaxVar\left(M\right)=Max\left(\left(\frac{1}{n}\right)\;UMM^{T}U\right)$$

Since $\frac{1}{n}\;MM^{T}$ is the same as the covariance matrix of *M*(*cov*(*M*)), *Var*(*M*) can be expressed, following:

(4) $$Var\;\left(M\right)=U^{T}cov\;\left(M\right)\;U$$

The Lagrangian function can be defined by performing the Lagrange multiplier method, following:

(5) $$L=U^{T}\;L=U^{T}cov\left(M\right)U-\delta \left(U^{T}U-1\right)$$

for (5), “*U^T^U*−1” is considered equal to zero since the weighting vector is a unit vector. Hence, the maximum value of *Var*(*M*) can be calculated by equating the derivative of the Lagrangian function (*L*), with respect to *U*, following:

(6) $$\frac{dL}{dU}=0$$

(7) cov(M)U−δU=(cov(M)−δI)U=0

where,

*δ*: eigenvalue of *cov*(*M*)
*U*: eigenvector of *cov*(*M*)

## 3. Results and Discussion

**Figure 1:**
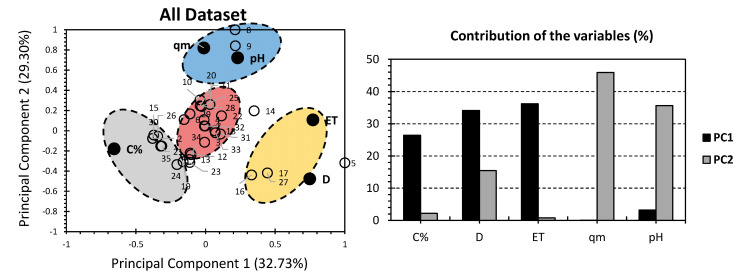
PCA for all datasets. Ref. [6] White bullets represent the 35 investigated graphene oxide hydrogels. Black bullets represent the adsorption properties involved. Different colors were used for clusters to make the interpretation of results easier.

Figure 1 shows the PCA biplot for the published results on the adsorption data of different composite hydrogels containing GO (and derivatives) used for the removal of dyes from water [6]. The first two PCs accounted for 62.03% of the total variance (32.73% for PC1 and 29.30% for PC2). The factors: C%, D, and ET, exhibited the highest contribution to PC1, accounting for 26.43%, 34.12%, and 36.22%, respectively. As for PC2, qm and pH accounted for the highest contributions, yielding 45.91% and 35.66% of the total contribution of these factors, respectively. The difference in factors’ contributions with respect to the investigated PCs indicates a high representation of the adsorption data of the investigated hydrogels. C% showed a negative influence on both PCs; however, it influenced PC2 to a lesser extent. For qm and pH, they presented certain proximity and were located on the top-right quarter of the biplot. More specifically, qm had a strong positive influence along PC2, with no influence along PC1. The factor pH had a slight positive influence along PC1 with a major positive effect along PC2. ET and D are located in the bottom-right corner of the biplot. More specifically, ET scored a strong positive influence along PC1, with no influence for PC2. For D, it scored a strong negative influence along both PCs.

PCA yielded four different distinguishable clusters of hydrogels: red, blue, yellow, and grey (Figure 1). It is quite interesting that the red cluster gathered most of the investigated hydrogels, indicating a poor to no influence of the studied factors on each hydrogel of this cluster. For the blue cluster, it gathered hydrogels 8 and 9 and showed a positive correlation along pH and qm. This indicates that alginate GO hydrogels (without polyvinyl alcohol) are more suitable for an elevated pH medium, and higher adsorption capacities are required. These findings are corroborated by Zhuang et al., where alginate GO hydrogels had the highest qm and the best tolerance for strong base [30]. For the yellow cluster, it gathered hydrogels 16, 17, and 27; and showed a positive correlation along ET and D. Since both 17 and 27 are the only sulfonated polyvinyl alcohol hydrogels, this could indicate that these hydrogels are better suitable for highly contaminated water. This is supported by Li and colleagues’ results, where both 17 and 27 scored the highest sorbent dosage D [32]. For the time to achieve equilibrium conditions (ET), the findings in hand could not confirm or inform its relevance to these hydrogels, as a part of the data is missing (Figure 1). For the grey cluster, it gathered hydrogels 10, 15, 21, 24, 26, 30, and 35 and showed a positive correlation with C%. This could generally indicate the relevance of the content of GO in composite hydrogels. No other findings can be depicted since the included hydrogels show significantly different functional groups and are, therefore, not similar.

**Figure 2:**
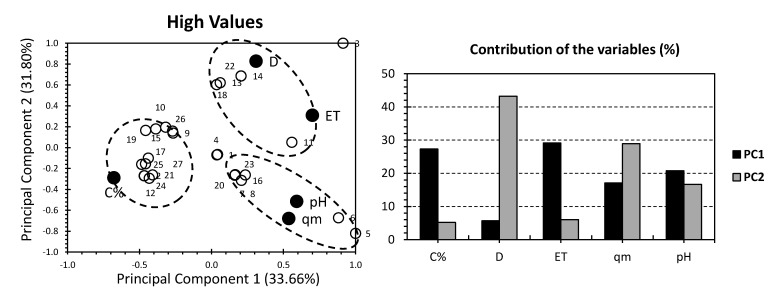
PCA for highly correlated values. Ref. [6] White bullets represent the 35 investigated graphene oxide hydrogels. Black bullets represent the adsorption properties involved.

**Figure 3:**
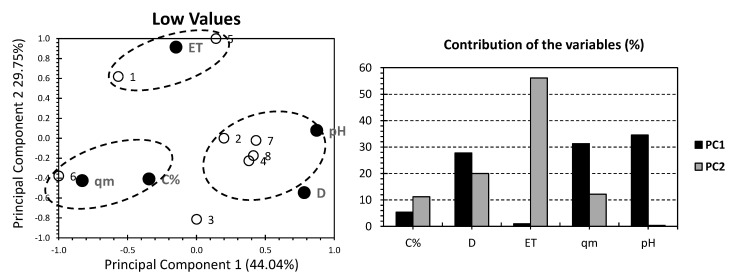
PCA for low correlated values. Ref. [6] White bullets represent the 35 investigated graphene oxide hydrogels. Black bullets represent the adsorption properties involved.

Even though most of the individuals have shown negligible influence by both PCs, all of the datasets for the PCA approach have shown quite interesting findings. Hence, ALG/GO hydrogels (without PVA) have shown more suitability for higher pH media and where higher adsorption capacities are required. PVA sulfonated hydrogels are estimated to be more likely applied where higher adsorbent doses (D) are required. To seek a better knowledge, the dataset will be split into: (a) high correlation individuals (having correlation factor, x > +0.2; Figure 2), (b) low correlation individuals (having correlation factor, −0.2 < x < +0.2; Figure 3).

Figure 2 shows the PCA biplot for the highly correlated individuals of the investigated GO hydrogels. The first two PCs accounted for 65.46% of the total variance (33.66% for PC1 and 31.80% for PC2; Figure 2) The slightly higher variance, if compared to the all-dataset approach (Figure 2) indicates that the following findings are more reliable than the total dataset PCA. For the factors, C and ET exhibited the highest contribution of PC1, accounting for 27.32% and 29.12%, respectively. As for PC2, D and qm accounted for the highest contributions, yielding 43.22% and 28.91% of the total contribution of this factor. Interestingly, both qm and pH showed moderate contributions along both PCs. Similar to the case of all datasets, C% in the PCA analysis showed a negative influence on both PCs. For qm and pH, they presented certain proximity and were located in the bottom-right quarter of the PCA-biplot. Therefore, qm and pH scored a strong positive and negative influence along PC1 and PC2, respectively (Figure 2). ET and D are located on the top-right of the PCA biplot. More specifically, D scored a strong and moderate influence along PC2 and PC1, respectively (Figure 2). ET and D are located in the top-right of the PCA biplot. More specifically, D scored a strong and moderate influence along PC2 and PC1, respectively (Figure 2) For ET, it scored a moderate influence on both PCs. %C was individually located on the bottom-left corner of the PCA biplot and presented a moderate negative influence along with both PCs. Even though the factors showed different distributions on the PCA biplot than the all-dataset approach, it revealed the same grouping. Additionally, a better distribution of the individuals is clear (Figure 2). This reveals the efficiency of dividing the dataset into high and low-correlation individuals (Figure 2 and Figure 3). In contrast, a high distribution of individuals makes seeking any relevant tendencies between hydrogels a rather tedious and time-consuming approach.

Figure 3 shows the PCA biplot for the low correlated individuals of the investigated GO hydrogels. The first two PCs accounted for 73.79% of the total variance (44.04% for PC1 and 29.75% for PC2; Figure 3). Once compared with the two previous approaches (Figure 1 and Figure 2), the higher variance of the low correlation individuals indicates that the following strategy is the most reliable one, as it copes with the highest amount of the “truth” in the investigated dataset. Factors, D, qm, and pH exhibited the largest contribution of PC1, accounting for 27.77%, 31.28%, and 34.56%, respectively. As for PC2, ET accounted for the highest contribution, yielding 56.15% of the total contribution of this factor. It is worth mentioning that different groupings were yielded than the two previous approaches (Figure 1 and Figure 2). Hence, ET is individually located on the upper part of the biplot, yielding a high positive influence following PC1 and a negligible one along PC2 (Figure 3). pH and D are located on the bottom-right quarter of the PCA biplot. More specifically, D scored strong positive and negative influences along PC1 and PC2, respectively. For pH, a strong positive influence along PC1, with a minor influence along PC, can be found. C% and qm are located in the bottom-left quarter of the PCA biplot. More specifically, qm scored a strong negative influence along with both PCs. For C%, it scored a moderate negative influence along with both PCs. For individuals, and similarly to the highly correlated individuals, it yielded multiple clusters containing hydrogels with very different matrices and functional groups, which prohibits any change of finding relevant findings between the hydrogels in hand.

## 4. Conclusions

This study aims to apply PCA to delineate interesting tendencies affecting the adsorption features of GO-based hydrogels. Different approaches were adopted, and each presented pros and cons. When PCA was run for the whole data set at once, ALG/GO hydrogels (without PVA) showed better tolerance in the basic medium and provided higher adsorption capacity to be implemented. PVA sulfonated hydrogels are considered preferably applied where higher adsorbent doses (D) are required.

Furthermore, we have attempted to develop a new strategy to reveal the outmost findings from the datasets. The adopted strategy involves splitting the individual hydrogels between high and low correlated ones. In our case, both groups of individual hydrogels showed a higher presentation of the total variance rather than having the total dataset analyzed all at once. Interestingly, the highest variance was yielded for the low correlated factors. This will allow a better seeking out of the tendencies between different hydrogels. Even though no specific trends were yielded when the various hydrogels were separated, the highest variance makes this method better suited for the provided data-driven study.

## Figures and Tables

**Table 1:** Adsorption data of different composite hydrogels containing graphene oxide (and derivatives) used for the removal of dyes from water (adapted with permission from Ref. [6]).

	Composite Hydrogel	C% ^a^	D ^b^	ET ^c^	qm ^d^	pH ^e^	References
1	PMPTC/GO	0.3	-	150	13		Wang et al. [23]
2	PAAm/GO	50	0.2	20	293		Yang et al. [24]
3	CMC/Aam/GO	10	4	720	185	6	Varaprasad et al. [25]
4	Chitin/TA/GO	7		400	231	7	Liu et al. [26]
5	CTS/GO			4000	10		Zhao et al. [27]
6	CTS/amino-functionalized-GO	20		5	385	7	Omidi and Kakanejadifard [28]
7	PVP/Aac/GO	0.2	5	40	78	7	Atyaa et al. [29]
8	Double ALG/GO network		1	1200	2300	8	Zhuang et al. [30]
9	Single ALG/GO network		1	1200	1800	8	Zhuang et al. [30]
10	Double ALG/PVA/GO network	5	0.1	480	1437	6	Kong et al. [31]
11	Single ALG/PVA/GO network	5	0.1	480	1256	6	Kong et al. [31]
12	ALG/immobilized GO network	5	0.2	200	181	5.4	Li et al. [32]
13	ALG/GO		5	60	122	5.3	Balkız et al. [33]
14	CTA/PAAc/GO	0.5	1	2250	297	7	Chang et al. [34]
15	CTS/GO	50	0.13	70	390	6.5	Chen et al. [35]
16	CTS/GO			50	3.5		Zhao et al. [27]
17	PVA/sulfonated-GO	1	80	720	5.1	6.2	Li et al. [32]
18	Cellulose/GO	0.5	20	20	123	7	Soleimani et al. [36]
19	Cellulose/GO	10	2	70	46		Liu et al. [26]
20	CMC/PVA/GO	0.7	1.5	80	89	8	Dai et al. [37]
21	*k*-CARR/GO	30		6	658	5.3	Yang et al. [38]
22	PEGDMA-rGO	1	2.5	720	60	7.4	Halouane et al. [39]
23	PAMm/GO	5		75	26		Thompson et al. [40]
24	PEGD/thiolated-GO	17		75	6		Liu et al. [26]
25	PAAc-*g*-XG/GO	0.5	0.25			7	Hosseini et al. [41]
26	PEI/GO			240	334		Guo et al. [42]
27	PVA/sulfonated-GO	1	80		4.4	6.2	Li et al. [32]
28	ALG/PAAc/Graphite			60	629	7	Verma et al. [43]
29	XG-*g*-PAAc/rGO	5	0.5	30	1052	6	Makhado et al. [44]
30	PAMm/GO	50	0.025	20	288		Yang et al. [24]
31	CTS/GO			250	1.9		Zhao et al. [27]
32	PMPTC/GO	0.3		150	12		Wang et al. [23]
33	Cellulose/GO	0.5	20	40	62	7	Soleimani et al. [36]
34	PEI/GO			240	132		Guo et al. [42]
35	ALG-Fe^3+^/rGO	50		360	18.4		Xiao et al. [45]

^a^ C% = Content of graphene oxide (and derivatives) (wt-%) in the composite hydrogel. ^b^ D = Adsorbent dosage (g/L). ^c^ ET = time necessary to achieve the equilibrium condition (min). ^d^ qm = Adsorption capacity (mg/g). ^e^ pH = potential of hydrogen is a scale used to specify the acidity or basicity of an aqueous solution.
